# Long-term effectiveness and cost-effectiveness of testing for alemtuzumab antidrug antibodies to guide treatment in multiple sclerosis: a modelling study

**DOI:** 10.1007/s10198-025-01854-8

**Published:** 2025-11-12

**Authors:** Timothy Jamieson, Florian Tomini, Sharmilee Gnanapavan, Borislava Mihaylova

**Affiliations:** 1 Health Economic and Policy Research Unit, Wolfson Institute of population Health, Queen Mary University of London, London, UK; 2 Blizard Institute, Barts and The London School of Medicine and Dentistry, Queen Mary University of London, London, UK; 3 Health Economics Research Centre, Nuffield Department of Population Health, University of Oxford, Oxford, UK

**Keywords:** Multiple sclerosis, Alemtuzumab, Anti-drug antibodies testing, Cost-effectiveness, Disease-modifying therapies

## Abstract

Biologic therapies are increasingly used in multiple sclerosis (MS), but often provoke anti-drug antibodies, potentially leading to treatment failure. Testing for anti-drug antibodies to guide treatment switching could improve clinical- and cost- effectiveness of MS treatment. We assess the value of testing for anti-drug antibodies to alemtuzumab, an effective but immunogenic MS therapy. We developed a microsimulation model to project disease progression, quality of life, and cost outcomes in people with relapsing-remitting MS initiating alemtuzumab treatment without and with alemtuzumab anti-body testing. Risk of anti-drug antibody development was informed by a UK cohort study of alemtuzumab-treated people with MS. UK guidance informed MS treatment strategies. Alemtuzumab anti-drug antibody test-directed treatment switching resulted in 0.02 fewer MS relapses per person; prolonged time to secondary progressive disease by 0.06 years; and yielded 0.02 additional years of life (0.06 Quality-Adjusted Life Years (QALY)). At £25/test, incremental cost per QALY gained was £47,861, with the additional cost arising from increased time on disease-modifying therapies (DMTs). Cost-effectiveness of anti-drug antibody testing was sensitive to anti-drug antibody development risk, their impact on drug efficacy, and costs of disease-modifying therapies (DMTs). Anti-drug antibody testing to inform MS treatment switching could improve clinical outcomes, but its cost-effectiveness depends on anti-drug antibody risk, its impact on drug efficacy, and costs of DMTs.

**Supplementary Information:**

The online version contains supplementary material available at 10.1007/s10198-025-01854-8.

## Introduction

Multiple sclerosis (MS) is an autoimmune disease of the central nervous system with immune-mediated destruction of the myelin covering of nerve cells leading to nerve degeneration and consequent disability [1]. MS is the commonest cause of non-traumatic disability in young adults worldwide [2]. The most common form of MS, relapsing-remitting MS (RRMS), seen in approximately 80% of people with MS (PwMS), has a disease course characterised by acute relapses of neurological symptoms followed by resolution, typically on a background of slowly worsening underlying disability over the long-term [1]. This is often followed by secondary progressive MS (SPMS) where acute relapses no longer occur, and progressive worsening of disability predominates [1].

Disease-modifying therapies (DMTs) approved for use in people with MS, particularly the relapsing-remitting form, have demonstrated effectiveness in reducing relapses and delaying progression of MS-related disability [3, 4]. However, suboptimal clinical responses to DMTs are a frequent challenge in the management of MS. Early detection of treatment failure and DMT switching is expected to improve outcomes [5].

DMTs in MS are increasingly based on recombinant human protein and monoclonal antibody technologies, ‘biologics’. These molecules show high propensity for provoking immune responses which may result in the development of anti-drug antibodies (ADAs), which have the potential to reduce clinical effectiveness, and likely contribute to hypersensitivity reactions [6, 7]. Testing of the immunogenicity of biologics is required for regulatory approval, but there is increasing interest in evaluating the clinical impact of immunogenicity in real world use since ADAs are seen to develop against most biologics [8, 9]. In the context of rheumatoid arthritis ADA testing is already used in clinical practice to direct use of some biologics [8].

Alemtuzumab, a highly effective biologic therapy approved for use in MS has been found to be particularly immunogenic, both amongst MS biologics and amongst biologics used in all disease areas [9]. Alemtuzumab is administered by intravenous infusion for 2 treatment cycles 12 months apart, followed by a further cycle in case of disease activity. In the pivotal alemtuzumab trials, CARE-MS I and CARE-MS II, 85% of individuals tested positive for ADAs after receiving the second alemtuzumab cycle [6, 9]. In some individuals the development of neutralising antibodies appears to reduce effectiveness resulting in clinical disease activity [10]. A recent cohort study in alemtuzumab-treated PwMS reported failure of effect on target immune cells in approximately 13% of people following the second alemtuzumab cycle, with high levels of neutralising ADAs found in the majority of these, and early indications of suboptimal clinical outcomes [7].

Testing for the development of ADAs may offer potential to predict treatment failure, avoiding the futile use of a costly therapy (most biologics used to treat MS, including alemtuzumab, cost over £20,000 per treatment year), and to enable timely switching to a more effective therapy [11].

The aim of our modelling study is to assess the cost-effectiveness of an ADA test-directed treatment switching strategy in people with RRMS treated with alemtuzumab compared to current practice without testing. Using a MS microsimulation model, we evaluate the effects on clinical and cost outcomes of using ADA testing to determine a further alemtuzumab cycle or a treatment switch when alemtuzumab-treated individuals experience recurrent disease activity and would therefore become eligible for a third alemtuzumab cycle.

## Methods

The structure of the MS model aligns with models submitted to the UK National Institute of Health and Care Excellence (NICE) technology appraisals committees. However, it also incorporates treatment sequencing, the absence of which is often considered a weakness in NICE appraisals [12]. We employ a microsimulation approach similar to Huygens and Versteegh (2021) to specifically examine treatment sequencing in MS [13]. No waning effect of treatment is included, with modelling of treatment failure instead taken from published data on treatment cessation and switching [14]. A lifetime horizon is chosen to reflect the long and variable disease course of MS over which the costs and benefits of DMTs are seen.

The MS model used in this analysis was an individual-level microsimulation model with a one-year cycle length and lifetime horizon from alemtuzumab initiation to death. The perspective adopted was that of the United Kingdom’s National Health Service. Health outcomes included the number of MS relapses, time to transition from RRMS to SPMS, life years and quality-adjusted life years. The quality-adjusted life years outcome captures both the duration and quality of life over the modelled time horizon. Costs included costs of DMTs, health and social care costs by MS-specific disability level, MS relapse-associated health and social care costs, and adverse event-related healthcare costs.

We report the incremental cost-effectiveness ratio of MS care with ADA testing compared to MS care without ADA testing for RRMS patients initiated on alemtuzumab treatment, expressed as the additional cost per quality-adjusted life year gained. In line with NICE health technology assessment guidance a 3.5% annual discount rate was applied to both cost and quality of life outcomes beyond the first year, to account for time preferences and the reduced present value of future costs and benefits [15].

### Study population

The target population is PwMS with RRMS initiating alemtuzumab treatment in the UK. We used the profiles of 21 RRMS patients initiating alemtuzumab in a tertiary neurology unit in Barts Health NHS Trust, London, UK, from 1 January 2021 to 31 March 2023. This period reflected contemporary UK prescribing practice in MS following a UK Medicines and Healthcare products Regulatory Agency safety update leading to more restricted prescribing [16]. Study population characteristics are described in Table 1 and the individual characteristics are provided in the supplementary material: supplementary methods.

**Table 1:** Baseline characteristics of the 21 MS patients initiating alemtuzumab

Characteristic	Mean (Range) or *N* (%)
Female sex	13 (62%)
Age at MS onset	31 (13–49)
Age at alemtuzumab initiation	37 (20–54)
EDSS at alemtuzumab initiation	3.5 (1–6)

*EDSS* expanded disability status scale, *MS* multiple sclerosis

### DMT use in MS

We modelled MS DMT treatment and decision-making based on the current NHS England treatment algorithm [4]. This includes three treatment lines, with a move to the next line being directed by ongoing disease activity, evidenced by the occurrence of an MS relapse, despite an adequate course of a DMT. Moving to the second treatment line allows a choice to be made from DMTs with higher impacts on relapse rates and disease progression but which may carry a more significant side-effect profile [3]. The third treatment line allows a switch to an alternative member of this group, excluding Ponesimod and Fingolimod [4]. A further distinction is made on the basis of the number of relapses in a year. More than one relapse in a twelve-month period defines rapidly evolving severe disease (RES), which has its own set of treatment options [4].

Alemtuzumab is allowed from the second line of treatment for RRMS with ongoing disease activity alongside Cladribine, Fingolimod and Ocrelizumab [4]. DMT switching is common in MS due to both drug intolerance and recurring disease activity [14]. In the presence of intolerance, switches are made within the treatment line an individual is on. In the presence of ongoing disease activity, evidenced by clinical relapse, a switch is made to one of the available options in the next treatment line; two or more relapses in an annual period direct a switch to the options available for rapidly evolving severe disease in the next treatment line [4]. We reflect the variability in clinical practice by giving equal likelihood of a switch being made to any of the relevant DMTs.

**Fig. 1:**
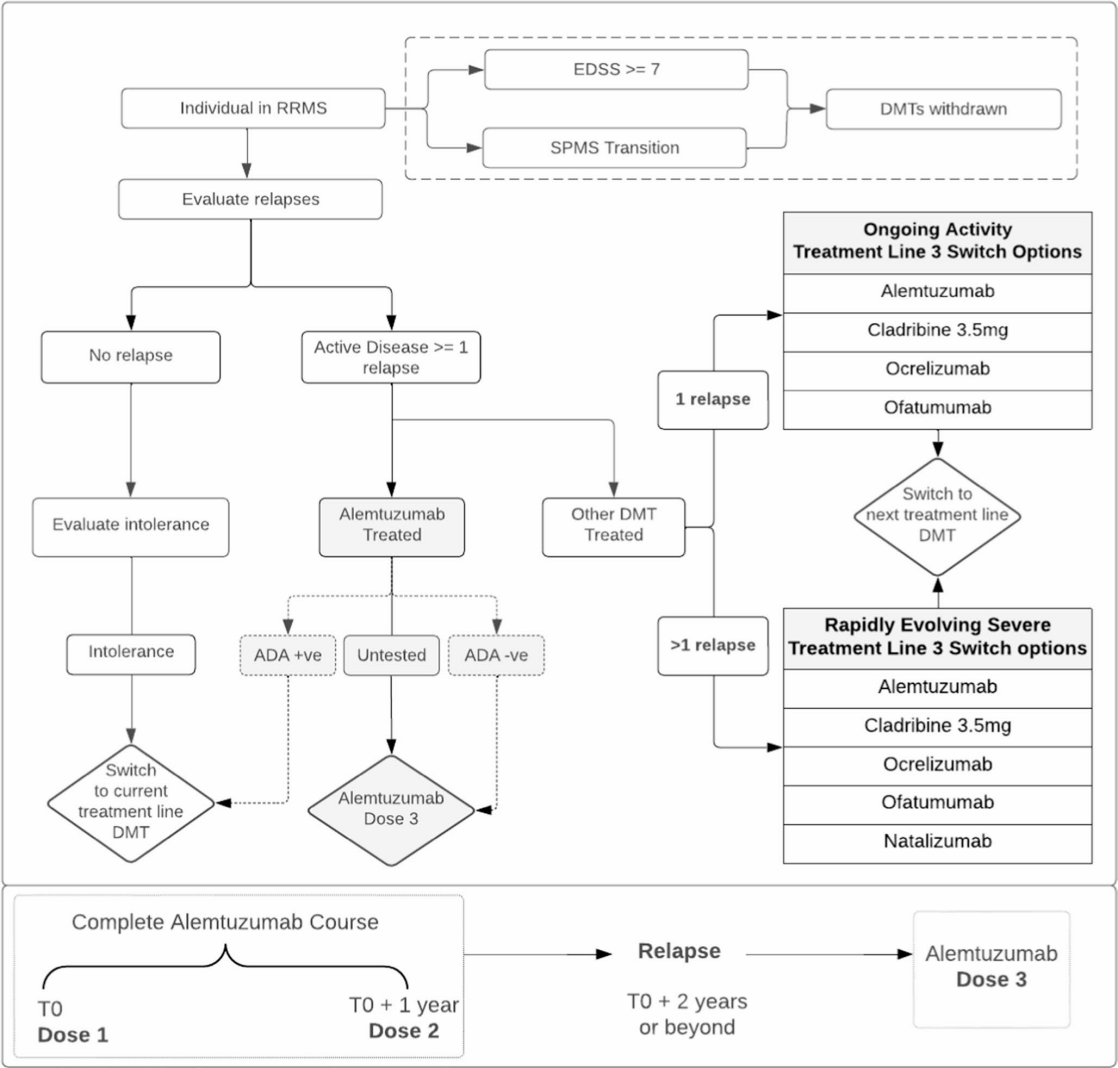
DMT Switching algorithm with alemtuzumab ADA testing and dose schedule

As per NHS England guidance, DMTs are withdrawn when an individual enters the secondary progressive form of MS or progresses to or beyond an Expanded Disability Status Scale (EDSS) score of 7 [4]. The EDSS is the most commonly used MS disease-specific disability rating scale, and is used clinically to monitor disease progression [17]. Figure 1 outlines the treatment and switching algorithm implemented.

### Alemtuzumab

Alemtuzumab is administered as 5 consecutive daily doses in year 1 (cycle 1), followed by 3 consecutive daily doses 12 months later (cycle 2) [18]. In the absence of ongoing disease activity, no further treatment is given (Fig. 1). A further three consecutive day infusion cycle may be administered if clinical disease activity occurs, or MRI imaging demonstrates relapse subsequent to the initial 2-year treatment [19].

There is evidence that patients with the highest anti-alemtuzumab antibody responses exhibit the poorest responses in terms of reducing lymphocyte numbers, which is the target of alemtuzumab, and there is emerging evidence of subsequent disease activity in those people [10]. Recent work examining a cohort of UK-based PwMS treated with alemtuzumab as part of a wider longitudinal trial found failure of lymphocyte depletion following a third alemtuzumab cycle in 13% of patients (4 out of 31). A number of the cohort received more than 3 doses, and among them 7 further failures of lymphocyte depletion were seen, with high ADA levels in 86% (6 out of 7). This informs the base case risk of alemtuzumab ADA development, of 0.11 (i.e. 13% x 86%; see supplementary material: supplementary methods for further details) [7].

### Specific adverse events included in modelling

As in previous modelling [13], two DMT-specific clinically important adverse effects were integrated in the model: (a) Autoimmune thyroid disease with alemtuzumab, and (b) Progressive Multifocal Leukoencephalopathy (PML) with natalizumab.

Alemtuzumab is associated with an excess risk of autoimmune thyroid disease, occurring in approximately one-third of those treated, at an average of 3 years following treatment initiation [20].

Natalizumab is associated with Progressive Multifocal Leukoencephalopathy (PML), occurring in between 0.1 and 10 cases per 1000 individuals, with risk related to both duration of natalizumab treatment and to the presence of antibodies against JCV [21]. Individuals experiencing PML have an approximate 25% risk of death and increased disability, reflected in an average 2-step increase in EDSS among those who survive [22]. Anti- JCV antibodies are modelled in the simulated population probabilistically using prevalence taken from the UK subpopulation of a Europe-wide study of JCV prevalence in PwMS (the JCV Epidemiology in MS trial) [23]. Subsequent risk of PML according to treatment duration and anti-JCV antibody presence is taken from a 2016 UK Medicines and Healthcare products Regulatory Agency safety update [21].

### Microsimulation model overview

**Fig. 2:**
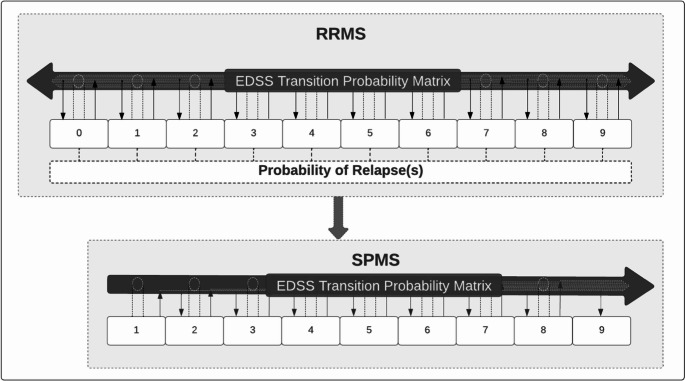
Multiple Sclerosis microsimulation model states EDSS: Expanded Disability Status Scale; RRMS: Relapsing-remitting multiple sclerosis; SPMS: Secondary Progressive multiple sclerosis

The model incorporates the trajectory of MS disease through RRMS and SPMS states (Fig. 2). In each annual period an individual who has not yet entered SPMS may experience relapse(s) and can transition to SPMS. Once an individual has entered SPMS it is assumed that relapses no longer occur alongside progression of disability.

Level of disability in each cycle is determined by the 0–9 integer valued MS-specific disability measure, EDSS, states an individual is in. The model uses whole-point EDSS states instead of the 0.5-point increments used clinically as evidence on disease progression, cost, and health-related utility is available at this level [24, 25]. In each cycle an individual may remain in their current EDSS state, or transition to another EDSS. In RRMS there is a possibility of movement to either higher or lower EDSS states, whilst in SPMS remaining in their current state or transitioning to a higher EDSS state are only possible. The probability of death in each annual period is modelled using UK general population age and sex-specific mortality risk adjusted for EDSS level (see supplementary material: supplementary methods) [26].

Input parameters determining microsimulation life course outcomes are described in the supplementary material: supplementary methods.

### Microsimulation study design

In the model, each individual in the study population is described by their sex, age, level of MS-related disability as measured by their EDSS at alemtuzumab initiation, age at MS onset, and functional system affected at MS onset (sensory vs. non-sensory). Information on sensory vs. non-sensory symptoms at onset was not available for the study population and was randomly assigned following a previously reported distribution (45% sensory; 55% non-sensory) [27].

Individuals enter the microsimulation process in RRMS with alemtuzumab initiated as a second-line therapy. In the first year in the model the first alemtuzumab cycle is given, followed by a subsequent cycle in the second year. On the basis of expert opinion that if ADAs develop, they are likely to develop shortly after administration of a second dose and will persist permanently, all individuals in the testing group have ADA levels assessed in the year of administration of the second dose with results to be used if a third dose is clinically indicated at any point thereafter.

If clinical relapse occurs at any time point beyond the initial alemtuzumab 2-year course, and a third alemtuzumab cycle becomes indicated, there is then a divergence between testing and no-testing strategies. ADA development is modelled in both groups and ADAs arise at the same rate. In the no-testing group the third dose is administered to all individuals. In the testing group those who are ADA test negative will have the third alemtuzumab dose administered, whilst a positive ADA test will direct a switch to another DMT in the same treatment line **(**Fig. 1**)**. In those individuals in whom alemtuzumab ADAs have arisen, it is assumed that alemtuzumab has no impact on rates of relapse or EDSS progression.

After either the third alemtuzumab cycle is given, or a switch is made to another treatment in line 2, on the basis of a positive ADA test in the testing group, routine care is applied to both groups.

### Uncertainty

Five hundred thousand microsimulations were executed for each individual profile to minimise uncertainty from random variability in the long-term disease course for each patient, at which number a < 1% difference in any outcome measure was seen with doubling of the number. In addition, to assess parameter uncertainty, probabilistic sensitivity analysis was undertaken with 300 parameter sets drawn for distributions of key model parameters determining the disease course, cost, and utility outcomes (parameters’ distributions are described in supplementary material: supplementary methods).

### Base case and scenario analyses

For base case analysis, we assumed ADA testing would not give false positive or false negative results, the risk of alemtuzumab ADAs was considered to be 0.11 (supplementary material: supplementary methods), and ADA presence was assumed to completely eliminate the clinical effect of alemtuzumab. Price per test was assumed to be £25, in line with a similar testing technology [28].

Scenario analyses were carried out to assess the impact of variation in key parameters, including the impact of varying DMTs price discounts from list price. This allows an assessment of sensitivity of results to undisclosed levels of price discount arising from confidential commercial agreements, as well as sensitivity to lower cost biosimilars becoming available.

### Software and computing

All analyses were performed using R (version 4.2.2). The model simulation utilised Queen Mary University of London’s Apocrita High Performance Computing facility, supported by Queen Mary University of London Research-IT [29].

## Results

Baseline characteristics of the 21 patients in the study population (Table 1) show a mean age at MS onset of 31 years, mean age at alemtuzumab initiation of 37 years; 62% female; and a mean EDSS at alemtuzumab initiation of 3.5, with a range of 1 to 6.

### Base case results

In the MS treatment pathway without ADA testing, the projected mean survival for the study population was 33.39 years (14.47 QALYs), with an estimated 0.99 relapses over their remaining lifetime. The average time to progression to SPMS was 12.9 years with £117,205 accumulated in DMT and MS-related health and social care costs over lifetime per patient (Table 2).

**Table 2:** Model-projected lifetime health outcomes and costs

Outcome	With alemtuzumab ADA testing Mean (SE)	Without ADA testing Mean (SE)		Difference Mean (SE) or Ratio (95% CI)
Duration on alemtuzumab (years)	9.15 (1.05)	9.32 (1.05)	−0.164 (0.007)	
Alemtuzumab Doses given (number)	2.11 (0.03)	2.16 (0.04)	−0.051 (0.002)	
Duration on any DMT (years)	12.38 (1.28)	12.33 (1.28)	0.051 (0.009)	
Health Outcomes				
Number of relapses	0.97 (0.14)	0.99 (0.15)	−0.020 (0.002)	
Duration to SPMS (years)	12.95 (1.16)	12.90 (1.16)	0.058 (0.009)	
Life-years	33.41 (0.95)	33.39 (0.95)	0.021 (0.004)	
Life-years discounted	19.19 (0.29)	19.18 (0.29)	0.006 (0.001)	
QALYs	**14.47 (1.31)**	**14.41 (1.31)**	**0.06 (0.009)**	
QALYs discounted*	**8.72 (0.62)**	**8.68 (0.62)**	**0.031 (0.004)**	
MS care costs (£)*				
Cost of ADA testing	£20.28 (£0.12)	0 (0)	£20.28 (£0.12)	
DMT Costs	£76,780 (£3,333)	£75,237 (£3,356)	£1,543 (£105)	
EDSS-related Costs	£41,363 (£5,318)	£41,438 (£5,333)	£−75.65 (£22.42)	
Relapse-related Costs	£209 (£34.32)	£213 (£34.82)	£−4.47 (£0.59)	
Thyroid Adverse Event Costs	£317 (£12.05)	£317 (£12.05)	£0.03 (£ 0)	
Total Cost	**£118**,**688 (£6**,**321)**	**£117**,**205 (£6**,**364)**	**£1**,**483 (£106)**	
Incremental Cost per QALY gained*	-	-	**£47**,**861 (£5,240)**	

*ADA* anti-drug antibody, *DMT d*isease-modifying therapy, *EDSS* expanded disability status scale, *QALY* quality-adjusted life year, *SE* standard error, *SPMS* secondary progressive multiple sclerosis

*All costs and QALYs beyond first year were discounted at 3.5% per year

Incorporating alemtuzumab ADA testing into the MS treatment pathway improved clinical outcomes in all endpoints: on average per patient there were 0.02 fewer relapses over a lifetime, time to SPMS was increased by 0.06 years, and survival was increased by 0.02 years (0.06 QALYs). Total lifetime costs with ADA testing were higher, with an average increase of £1,483 per patient. Although costs arising from MS-related disability and those associated with relapse were lower (by £76 for MS-related disability and £4.50 for relapse), the increased use of DMTs, of 0.05 treatment years on average, led to an additional DMT cost of £1,543 per patient. The incremental cost per QALY with ADA testing was £47,861.

The outcomes varied across the 21 study participants with the ADA testing strategy demonstrating consistently better clinical outcomes, but at increased costs (Table 3 and supplementary material: Supplemental Table 1). Incremental cost-effectiveness ranged from £43,818 to £92,818 per QALY. Health outcomes were most closely associated with EDSS level at alemtuzumab initiation, with patients at lower EDSS levels seeing greater health benefits (a maximum of 0.059 QALYs (discounted) seen in an individual with EDSS 1). Conversely, the additional cost seen in the testing arm decreased with higher EDSS level, due to much lower lifetime DMT costs (minimum difference in costs of £414 (discounted) in an individual with EDSS 6).

**Table 3:** Sensitivity analyses of cost-effectiveness of alemtuzumab ADA testing

Parameter	Parameter Value (Range)	Incremental Cost/QALY gained (Range)*
Risk of alemtuzumab ADA development	1–20% risk	£54,346 - £47,523
Reduction in alemtuzumab effectiveness in presence of ADA	0–100% reduction	See Fig. 3
ADA test false positive rate	0–10%	£47,861 - £238,549
ADA test false negative rate	0–10%	£47,861 - £47,739
ADA test cost	£5 - £500	£47,218 – £60,138
Price Discount for all DMTs	0%, 25%, 50%, 75%	£47,861; £35,299; £23,511; £11,722

*ADA* anti-drug antibody, *DMT* disease-modifying therapy, *QALY* quality-adjusted life year

*With costs and QALYs beyond first year discounted at 3.5% per year

### Scenario and sensitivity analysis

**Fig. 3:**
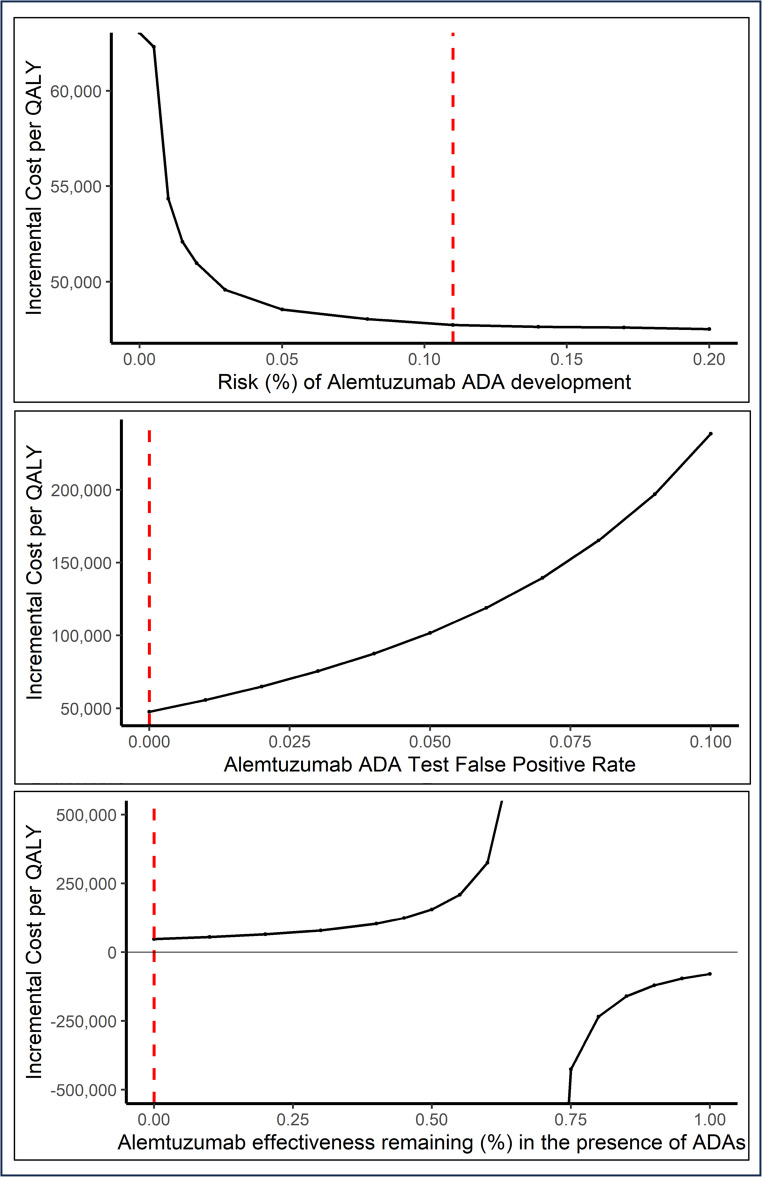
Results of key sensitivity analyses of cost-effectiveness of alemtuzumab ADA testing

Scenario analyses indicated that cost-effectiveness remained stable down to about 5% risk of ADA development (Fig. 3). Cost-effectiveness was most sensitive to the impact of ADAs on alemtuzumab effectiveness and test false positive rates. If the remaining effectiveness of alemtuzumab in the presence of ADAs was 75% or higher, there is no value of testing for ADAs (Fig. 3). Similarly, with a false positive rate of 2%, the incremental cost-effectiveness rose to about £65,000/QALY; false negative rates in a range of 0–10% however did not impact the cost-effectiveness (Table 3).

If a 25% discount is applied to all DMT list prices, the incremental cost per QALY gained with alemtuzumab ADA testing reduced to £35,299/QALY, and with 75% discount reduced further to £11,722/QALY (Table 3).

## DISCUSSION

The study finds that using alemtuzumab ADA testing to direct treatment switching decisions in MS could improve clinical outcomes by reducing number of relapses, increasing time to secondary progressive disease and improving quality of life-adjusted survival for PwMS. The cost-effectiveness of this strategy, however, importantly depends on test characteristics (particularly the rate of test false positives), and costs of DMTs.

Although the incremental cost-effectiveness ratio we report for alemtuzumab ADA testing is higher than the £20,000 to £30,000/QALY range typically considered cost-effective by UK decision-makers [15, 30, 31], the current NICE guidance recommends the application of severity modifiers for high QALY shortfall conditions [15]. The application of a severity modifier of 1.2, likely to be applicable in MS, gives a denominator of 0.0372 QALYs (discounted), reducing incremental cost-effectiveness to £42,559/QALY [30]. The commercially privileged price agreements in place for many MS DMTs may include 25% discounts, in which case the incremental cost-effectiveness ratio would become £29,510/QALY with the severity modifier applied, which is within the acceptable cost-effective range. Aside from DMT costs, two parameters are found to be particularly important: ADA test false positive rate and the impact of ADAs on alemtuzumab’s efficacy. Both could affect the relatively higher effectiveness of alemtuzumab compared with the alternatives to which a switch may be made, which are, on average, 75% as effective [3]. If ADAs do not reduce alemtuzumab efficacy beyond this, then a switch becomes a switch to inferior treatment and is clinically suboptimal. False positive test results carry the same consequence.

There is more established evidence in several disease areas, particularly inflammatory bowel disorders and inflammatory joint conditions, that ADAs to biologic therapies can impact efficacy and increase rates of infusion reactions, but significant variability between biologics and between methods of testing for ADAs exist [32, 33]. Testing for ADAs as part of treatment decision-making in these disease contexts has been demonstrated to provide clinical benefit, but economic evaluation literature is sparse, and to our knowledge this is the first evaluation in the context of MS [34–36]. Published economic evaluations in rheumatoid arthritis and inflammatory bowel disease have found clinical benefit alongside neutral to cost saving impacts on resources, but have been undertaken with short time horizons and with modelling approaches less well suited to the complex and long-term disease courses seen in MS [33, 35–38]. In contrast using a lifetime individual-level microsimulation we report clinical benefit but do not find cost savings. This is explained in particular by the extra costs arising from longer administration of other DMTs, which is enabled by the improved clinical outcome of delayed progression to SPMS, after which point DMTs are withdrawn. This additional cost reflects the high cost of alternative DMT options when a switch is made, rather than the cost of the testing technology itself.

Alemtuzumab is unusual in having front-loaded costs due to its dosing method – a full treatment course of alemtuzumab is administered as two infusion cycles completed in two years, other DMTs have similarly high annual cost but on an ongoing annual basis. Alemtuzumab is also particularly immunogenic compared to other DMTs. Extending this study to other DMTs would therefore be valuable. Although immunogenicity with other DMTs may be lower, the cost and relative efficacy of a particular DMT compared to other DMTs will impact significantly on effectiveness and cost-effectiveness of any switching strategy.

There is a significant need for both technical work in specifying and measuring ADAs, and for longitudinal series determining parameters relating to ADA development risk and their impact on efficacy of DMTs to inform evaluations of clinical and cost effectiveness. As biologic therapies become more widely used, understanding individual variability in response is likely to become more important.

Our study’s strengths lie in the combination of flexible individual-level microsimulation modelling of real-world disease trajectories and treatment pathways, with modern computational capability. This allowed us to resolve uncertainty arising from underlying variability in the individual disease course and to robustly assess parameter uncertainty. Our results provide insight into the potential value of ADA testing to optimise treatment pathways in MS, but some limitations must be highlighted. Firstly, the risk of ADA development is based on small number of patients from a single cohort study, and we have had to make assumptions on the clinical impact of ADAs on the basis of the surrogate marker measured of lymphocyte depletion [7]. Secondly, the population of PwMS modelled, although from a centre with a large number of PwMS under its care, may be limited in generalisability to other centres and other countries. Alemtuzumab eligibility criteria are standardised in UK NHS guidance, and in the European Union alemtuzumab is similarly authorised for use in active MS beyond the first DMT, but significant variation is seen in clinical practice in DMT choice and use [39]. It is also of note that with a trend towards earlier use of ‘high-efficacy’ DMTs, which include alemtuzumab, future practice may trend towards increased use particularly in those at lower EDSS levels [40]. Thirdly, our cost-effectiveness findings are most relevant to the UK since we use UK data for costs of DMTs and health and social care costs of MS patients. Finally, although scenario analysis explored the impact of test characteristics, and cost-effectiveness can be forecast from this analysis, inclusion of evidence-based test characteristics is required to produce more robust cost-effectiveness estimates.

In conclusion, we find that testing of alemtuzumab ADAs to guide treatment switching in MS has the potential to improve health outcomes cost-effectively. The evaluative framework we have developed, made available with this study, is adaptable to any biologic therapy in MS where ADAs may have impact and highlights key areas for evidence gathering. The analytical approach and broader findings of our study are likely to extrapolate to other disease contexts where biologic therapies may be applied.

## Supplementary Information

Below is the link to the electronic supplementary material.

Supplementary Material 1(DOCX 155 KB)

## Data Availability

All data is reported in manuscript. The R code of the MS model (License: MIT License) is available from https://github.com/TDJamieson/MSBiologicAntiDrugAntibodyModelling
