# Supplementary material for: Long-term effectiveness and cost-effectiveness of testing for alemtuzumab antidrug antibodies to guide treatment in multiple sclerosis: a modelling study
Source: Eur J Health Econ. 2025 Nov 12;27(4):869–79. doi: 10.1007/s10198-025-01854-8 (PMC13350217; doi:10.1007/s10198-025-01854-8)
Supplement: Supplementary file 1 — Supplementary Material 1(DOCX 155 KB) [file 10198_2025_1854_MOESM1_ESM.docx]

**European Journal of Health Economics**

**Long-term effectiveness and cost-effectiveness of testing for alemtuzumab anti-drug antibodies to guide treatment in multiple sclerosis: a modelling study**

**Supplementary Material**

**AUTHORS**

Timothy Jamieson^1^; Florian Tomini^1^; Sharmilee Gnanapavan^2^; Borislava Mihaylova^1,3^

^1^Health Economic and Policy Research Unit, Wolfson Institute of population Health, Queen Mary University of London, UK

^2^Blizard Institute, Barts and The London School of Medicine and Dentistry, Queen

Mary University of London, London, UK

^3^Health Economics Research Centre, Nuffield Department of Population Health,

University of Oxford, Oxford, UK

**CORRESPONDING AUTHOR**

Timothy Jamieson, Health Economic and Policy Research Unit, Queen Mary

University of London, Yvonne Carter Building, 58 Turner Street, London E1 2AB,

UK. t.jamieson@qmul.ac.uk

# Contents

[Contents 1](#_Toc205367351)

[Supplementary methods 2](#_Toc205367352)

[1. Microsimulation model inputs – disease and life course 2](#_Toc205367353)

[2. Microsimulation model inputs – cost and quality of life 4](#_Toc205367354)

[3. Mortality risk 5](#_Toc205367355)

[4. Annual relapse risk 6](#_Toc205367356)

[5. Risk of DMT intolerance 7](#_Toc205367357)

[6. Annual cost of disease-modifying therapy 8](#_Toc205367358)

[7. Annual health and social care costs (UK £), by EDSS and number of relapses 9](#_Toc205367359)

[8. Costs of management of autoimmune thyroid disease 10](#_Toc205367360)

[9. Risk of inhibitory anti-alemtuzumab antibodies 11](#_Toc205367361)

[10. Parameters subject to probabilistic sensitivity analysis (PSA) with distributions used 12](#_Toc205367362)

[11. Characteristics of Study Population 13](#_Toc205367363)

[Supplemental Table 1. Model-projected incremental outcomes for individual study participants with alemtuzumab ADA testing strategy 14](#_Toc205367364)

# Supplementary methods

## 1. Microsimulation model inputs – disease and life course

| **Component** | **Description of source data** |
| --- | --- |
| **Transition between Expanded Disability Status Scale (EDSS) states in relapsing-remitting multiple sclerosis (MS)** | Transition probability matrices for transitions between EDSS states 0-9 for people in the relapsing-remitting MS disease state were sourced from the British Columbia MS dataset, Palace et al., 2014 [1], with separate transition probability matrices for people with MS onset at under 28 years of age and with MS onset at over 28 years of age which was identified as the only significant determinant of progression probability. |
| **Transition between EDSS states in secondary progressive MS** | The transition probability matrix for transitions between EDSS states 1-9 for people who had transitioned to the secondary progressive MS disease were sourced from Mauskopf et al., 2016 where aggregated evidence from the London Ontario MS database and DEFINE and CONFIRM trials was used to produce a single transition probability matrix for secondary progressive MS [2]. There was zero probability of transition to any lower EDSS state from a given EDSS state. |
| **Transition from relapsing-remitting MS to secondary progressive MS** | Transition probabilities for transition from relapsing-remitting MS to secondary progressive MS for each EDSS level were also sourced from Mauskopf et al., 2016 [2]. These were derived from time-to-SPMS data in the London Ontario database, and based on data presented by Scalfari et al., 2010 [3]. |
| **Mortality risk** | Background UK age- and sex-specific mortality risk was sourced from UK Office for National Statistics life tables (mortality rates for 2021) [4]. These baseline risks were adjusted for EDSS, with EDSS-specific mortality risk multipliers informed from Zimmermann et al. 2018 [5]. The EDSS-specific multiplier values and derived uncertainty parameters are presented in **supplementary methods section 3**. |
| **Annualised relapse rate in relapsing-remitting MS** | Relapse rates for people in relapsing-remitting MS are dependent on their sex, age at onset of MS, type of symptoms at onset – sensory vs non-sensory, and MS disease duration, which is updated in each annual cycle.  Individual and cycle-specific relapse rates were simulated based on these characteristics using am unpublished quasipoisson regression model (**supplementary methods section 4**) undertaken as part of work by Tremlett et al., 2008, exploring the impact of age and time since onset of MS on relapse rates in the British Columbia MS dataset [6]. These rates were used to parameterise a poisson distribution in each cycle from which a random draw determines the occurrence of relapse(s) in that cycle. |

| **Component** | **Description of source data** |
| --- | --- |
| **Autoimmune thyroid disease risk** | The development of autoimmune thyroid disease in people on alemtuzumab was modelled once, at 3 years following the first dose of the initial course.  Risk of autoimmune thyroid disease was informed by a systematic review by Scappaticcio et al., 2020, that reported the proportion of alemtuzumab-treated individuals developing autoimmune thyroid disease in a total of 1362 patients in 7 included studies [7]. |
| **Progressive Multifocal Leukoencephalopathy (PML) risk** | The risk of developing PML in people on Natalizumab was dependent on the presence of anti-JCV antibodies (which imply the presence of inactive John Cunningham Virus (JCV), and which may be reactivated and may result in PML), and the duration of Natalizumab treatment. The PML risk was informed from a Medicines and Healthcare products Regulatory Agency update, 2016 [8].  Whether or not an individual was anti-JCV antibody positive was modelled at the start of microsimulation, with random assignment according to the proportion who were found to be anti-JCV positive in the UK subset of the JCV Epidemiology in MS (JEMS) trial [9]. |
| **PML morbidity and mortality** | The subsequent risk of mortality in people who experience PML was sourced from a 2015 review of 372 patients on Natalizumab who experienced PML which reported about 25% mortality [10].  The impact of PML on disability was taken from the same source, which reported an average two-step increase in EDSS in PML survivors [10]. |
| **Disease-modifying therapy impact on annualised relapse rate** | Disease-modifying therapy impact on both the incidence rate ratio of relapses, and the cumulative disability progression, were sourced from a network meta-analysis by Hennessy et al., 2022 [11]. That study was undertaken to characterise Ponesimod efficacy. This was assessed at 12 weeks by Hennessy et al.; relative risk was assumed constant over the annual cycle modelled. |
| **Disease-modifying therapy impact on EDSS progression** |  |
| **Intolerance to disease-modifying therapies** | The annual risk of stopping a disease-modifying therapy due to intolerance or adverse events was derived from work based on the European Big Multiple Sclerosis Data Network [12]. That study examined the incidence of stopping in 269,822 treatment episodes in 110,326 patients from 1997 to 2016. The derivation of DMT-specific stopping rates specifically arising from adverse events and intolerance is shown in **supplementary methods section 5**. |

| **Component** | **Description of source data** |
| --- | --- |
| **Disease-modifying therapy costs** | Annual costs for disease modifying therapy were calculated using dose prices listed in the British National Formulary combined with information on number of doses required and administration methods at medicines.org.uk [13, 14]. |
| **Health and Social Care costs** | EDSS-related and relapse-related health and social care resource costs were sourced from the South-West Impact of Multiple Sclerosis Project, a regional UK longitudinal survey including 1441 people with MS who self-reported health and social care use 2004-12 [15]. Costs were inflated to 2021. No data was available from this source for EDSS 9 due to an insufficient sample size, so a multiplier was derived from a publication from the European MS network [16]. Costs used and their derivation are given in **supplementary methods section 7**. |
| **Autoimmune thyroid disease costs** | The costs of investigating and managing autoimmune thyroid disease in the proportion of alemtuzumab-treated individuals who develop it are reported in **supplementary methods section 8**. It uses a weighted mean cost for the estimated costs of investigation and management of Grave’s Disease and Hashimoto’s thyroiditis, the two most common types of autoimmune thyroid disease seen. |
| **MS-related Quality of Life utility values** | Quality of life utility in each cycle was based on an individual’s sex, age, time since diagnosis of MS, MS type (RRMS vs SPMS), and current EDSS in that cycle. Utilities were informed from a linear regression model in a UK-based cohort of 14,385 people with MS who provided EQ-5D-3L responses between 2011 and 2019 [17].  A single disutility was applied if one or more relapses are experienced in a cycle. This value was informed by an earlier study by the same group in the South-West Impact of Multiple Sclerosis Project who produced the health and social cost information above. Here, self-reported EQ-5D-3L, outcomes were evaluated [18]. A disutility of -0.076 (SD 0.009) was applied if 1 or more relapses occur, the difference in the presented mean utility for those with no relapses in the prior 6 months and those with one or more relapses [18]. |

## 2. Microsimulation model inputs – cost and quality of life

## 3. Mortality risk

| **EDSS** | **Category** | **Multiplier (Confidence Interval)** |
| --- | --- | --- |
| 0 | - | 1 (1, 1) |
| 1 | Mild | 1.43 (0.89, 2.02) |
| 2 | Mild | 1.6 (1.06, 2.35) |
| 3 | Mild | 1.64 (1.09, 2.42) |
| 4 | Mild | 1.67 (1.13, 2.49) |
| 5 | Moderate | 1.84 (1.37, 2.92) |
| 6 | Moderate | 2.27 (1.8, 3.77) |
| 7 | Moderate | 3.1 (2.62, 5.38) |
| 8 | Severe | 4.45 (2.67, 6.14) |
| 9 | Severe | 6.45 (4.68, 10.07) |

As described in **supplementary methods section 1,** the risk of mortality in each cycle was based on background UK population mortality risk for an individual’s age in that cycle and sex, and this baseline risk was modified by an EDSS-specific mortality multiplier shown above. These multipliers were sourced from US-ICER work based on mortality by MS-related disability level in 2348 Canadian MS clinic patients [4, 5, 19, 20]. The multiplier was generated using the following equation:

$${Multiplier}_{EDSS}=0.0219 \times{EDSS}^{3}-0.1972 \times{EDSS}^{2}+0.6069 \times EDSS+1$$

This equation was sourced from the supplementary material in Zimmermann et al., 2018, who derived this from data presented in Pokorski et al., 1997, presented below [20]. Mortality multipliers generated by this equation for EDSS 5 and 8 are seen at the boundaries of the moderate and severe categories. Confidence intervals for each of these three categories were derived from the numbers of observed and expected deaths. EDSS-specific multipliers for the EDSS values falling into each category had these standard errors applied in order to parameterise uncertainty in probabilistic sensitivity analysis.

| **Category** | **Observed** | **Expected** | **Mortality Ratio** | **Standard Error** |
| --- | --- | --- | --- | --- |
| Mild | 33 | 20.7 | 1.60 | 0.278 |
| Moderate | 58 | 31.5 | 1.84 | 0.242 |
| Severe | 24 | 5.4 | 4.44 | 0.907 |

## 4. Annual relapse risk

Results of quasipoisson regression of annualised relapse rates on relevant predictors in the British Columbia MS dataset, undertaken but not included in published output by Tremlett et al., 2008 was used (personal communication with Helen Tremlett and Yinshan Zhao) [6]**.** Onset age and MS duration were interacted. NB types of symptoms at onset were aggregated by us (Jamieson et al.) into sensory vs non-sensory, since this was the only statistically significant symptom-related predictor.

| **Variable** | **Coefficient (Std. Error)** |
| --- | --- |
| (Intercept) | -1.511 (0.086) |
| Female Sex | 0.141 (0.037) |
| Male Sex (reference) | 0 |
| Sensory symptoms at MS onset | 0.177 (0.045) |
| Non-sensory symptoms at MS onset (reference) | 0 |
| Onset age >40 * MS duration >30 years | -2.371 (1.056) |
| Onset age >40 * MS duration 26-30 years | -1.578 (0.504) |
| Onset age >40 * MS duration 21-25 years | -1.493 (0.358) |
| Onset age >40 * MS duration 16-20 years | -0.928 (0.166) |
| Onset age >40 * MS duration 11-15 years | -0.56 (0.132) |
| Onset age >40 * MS duration 6-10 years | -0.278 (0.12) |
| Onset age >40 * MS duration 0-5 years | 0.137 (0.1) |
| Onset age 30-39 * MS duration >30 years | -1.636 (0.243) |
| Onset age 30-39 * MS duration 26-30 years | -1.3 (0.221) |
| Onset age 30-39 * MS duration 21-25 years | -0.799 (0.131) |
| Onset age 30-39 * MS duration 16-20 years | -0.421 (0.106) |
| Onset age 30-39 * MS duration 11-15 years | -0.213 (0.099) |
| Onset age 30-39 * MS duration 6-10 years | -0.123 (0.092) |
| Onset age 30-39 * MS duration 0-5 years | 0.289 (0.088) |
| Onset age 20-29 * MS duration >30 years | -1.122 (0.144) |
| Onset age 20-29 * MS duration 26-30 years | -0.758 (0.123) |
| Onset age 20-29 * MS duration 21-25 years | -0.559 (0.108) |
| Onset age 20-29 * MS duration 16-20 years | -0.199 (0.093) |
| Onset age 20-29 * MS duration 11-15 years | -0.131 (0.088) |
| Onset age 20-29 * MS duration 6-10 years | 0.099 (0.086) |
| Onset age 20-29 * MS duration 0-5 years | 0.239 (0.084) |
| Onset age <20 * MS duration >30 years | -0.715 (0.183) |
| Onset age <20 * MS duration 26-30 years | -0.471 (0.161) |
| Onset age <20 * MS duration 21-25 years | -0.303 (0.137) |
| Onset age <20 * MS duration 16-20 years | -0.158 (0.115) |
| Onset age <20 * MS duration 11-15 years | -0.064 (0.109) |
| Onset age <20 * MS duration 6-10 years | -0.14 (0.087) |
| Onset age <20 * MS duration 0-5 years (reference) | 0 |
| (Scale) | 2.668 |

## 5. Risk of DMT intolerance

| **Name** | **Stopping rate** | **Annual probability of intolerance** |
| --- | --- | --- |
| Placebo | 0 | 0 |
| Alemtuzumab | 0.229 | 0.0683 |
| Cladribine 3.5mg | 0.229 | 0.0683 |
| Cladribine 5.25mg | 0.229 | 0.0683 |
| Daclizumab | 0.229 | 0.0683 |
| Dimethyl fumarate | 0.229 | 0.0683 |
| Fingolimod | 0.197 | 0.0589 |
| Glatiramer 20mg | 0.258 | 0.0771 |
| Glatiramer 40mg | 0.258 | 0.0771 |
| IFN B-1A 22mcg SC | 0.233 | 0.0697 |
| IFN B-1A 30mcg IM | 0.233 | 0.0697 |
| IFN B-1A 44mcg SC | 0.233 | 0.0697 |
| IFN B-1B | 0.233 | 0.0697 |
| Natalizumab | 0.226 | 0.0676 |
| Ocrelizumab | 0.229 | 0.0683 |
| PEG B-1A | 0.229 | 0.0683 |
| Rituximab | 0.229 | 0.0683 |
| Teriflunomide 14mg | 0.229 | 0.0683 |
| Teriflunomide 7mg | 0.229 | 0.0683 |
| Ofatumumab | 0.229 | 0.0683 |
| Diroximel fumarate | 0.229 | 0.0683 |
| Ponesimod | 0.229 | 0.0683 |
| **Mean stopping rate across DMTs:** | | 0.229 |
| **Proportion with intolerance or adverse event as switch reason:** | | 0.299 |

Hillert et al., 2021, presented specific overall stopping rates per person-year from the Big MS Data Network for Fingolimod, Glatiramer, Interferon-beta, and Natalizumab, and also the stopping rate averaged over all DMTs [12].

Intolerance was cited as the stopping reason for 16.1% of stops, and adverse events the stopping reason for 13.8% stops. These reasons were combined to give an intolerance/adverse event stopping reason in 29.9% of stops, a proportion of 0.299.

The overall stopping rate for each DMT was multiplied by this proportion to give an estimate of annual stopping probability specifically secondary to intolerance or adverse event. If a DMT did not have a stopping rate specifically given, its stopping rate was taken to be the average stopping rate across all DMTs (0.229).

## 6. Annual cost of disease-modifying therapy

Annual DMT costs were based on British National Formulary prices with the number of doses necessary for an annual course incorporated. Cladribine and alemtuzumab are given in short, separated cycles and are costed by year of administration; other DMTs are given continuously over time [13, 14].

| **Name** | **Annual cost (UK£)** |
| --- | --- |
| No treatment | £ 0 |
| Alemtuzumab cycle year 1 | £ 35,225.00 |
| Alemtuzumab cycle year 2 | £ 21,135.00 |
| Alemtuzumab cycle 3 | £ 21,135.00 |
| Natalizumab | £ 14,690.00 |
| Ocrelizumab | £ 9,580.00 |
| Cladribine 3.5mg cycle Year 1 | £ 14,330.70 |
| Cladribine 3.5mg cycle Year 2 | £ 14,330.70 |
| Fingolimod | £ 19,110.00 |
| Dimethyl fumarate | £ 17,849.00 |
| Glatiramer 20mg | £ 6,681.35 |
| IFN B-1A 44mcg SC | £ 14,095.61 |
| IFN B-1B | £ 7,260.99 |
| Teriflunomide 14mg | £ 13,491.92 |
| IFN B-1A 22mcg SC | £ 10,634.34 |
| IFN B-1A 30mcg IM | £ 8,502.00 |

## 7. Annual health and social care costs (UK £), by EDSS and number of relapses

| **EDSS** | **Mean annual cost (SE)** |
| --- | --- |
| 0 | £1182 (651) |
| 1 | £1055 (390) |
| 2 | £830 (213) |
| 3 | £774 (187) |
| 4 | £1162 (256) |
| 5 | £1166 (278) |
| 6 | £1512 (218) |
| 7 | £1526 (418) |
| 8 | £3849 (916) |
| 9 | £4426 (1222) |

No cost for EDSS 9 was available from Hawton & Green, 2016, due to insufficient sample size at that EDSS. A multiplier, of 1.15, was applied, based on the ratio between costs at EDSS 8 and 9 in an alternative source of costs from the European MS network [15, 16]. The population included in the European MS network was on average much older than the UK MS population so specific costs from this study were not taken but an assumption was made that the ratio of costs between EDSS 8 and 9 will be similar. All costs presented in Hawton & Green, 2016, were 6-month costs and were therefore doubled to correspond to annual costs. We also inflate from 2012 to 2021 values.

| **Number of relapses** | **Mean annual cost (SE)** |
| --- | --- |
| 0 | £0 |
| 1 | £262 |
| 2 | £353 |
| 3 | £267 |
| 4 | £405 |

Cost of a relapse was also sourced from Hawton & Green, 2016 [15]. The study reported an overall mean health and social cost of £229 if no relapses were experienced. It was assumed that this was the background average EDSS-related cost, so here we take the cost for each number of relapses net of this, giving the additional cost relative to that cost if no relapses are experienced. These are again doubled to 12-month costs and inflated to year 2021 as per the EDSS-related costs above.

## 8. Costs of management of autoimmune thyroid disease

The cost of managing adverse events was sourced from NHS England unit costs for the investigations and treatments required in their management [21, 22]. Two types of autoimmune thyroid disease arise most commonly – Grave’s Disease and Hashimoto’s thyroiditis [7].

It was assumed that Grave’s disease was treated with radioiodine ablation of the thyroid followed by lifelong thyroid hormone replacement, due to the instability of thyroid hormone levels in alemtuzumab-induced Grave’s disease [23, 24].

Hashimoto’s thyroiditis was assumed to require lifelong thyroid hormone replacement.

The weighted mean cost across conditions was estimated from the individual disease management costs combined with the proportion of autoimmune thyroid disease they represent, informed from Scappaticcio et al., 2020 [7].

|  | **Graves management (UK £)** | | | | |
| --- | --- | --- | --- | --- | --- |
| Year | Total | Endocrinology OP attendance | RI ablation | Thyroxine 28 tabs x 13 | TFTs x 2 Y1-2; x1 3+ |
| 1 | £1,275.73 | £330.26 | £930.72 | £11.05 | £3.70 |
| 2+ | £14.75 | £0.00 | £0.00 | £11.05 | £3.70 |

|  | **Hashimoto management (UK £)** | | | | |  |
| --- | --- | --- | --- | --- | --- | --- |
| Year | | Total | Endocrinology OP attendance | Thyroxine 28 tabs x 13 | TFTs x 2 Y1-2; x1 3+ | |
| 1 | | £345.01 | £330.26 | £11.05 | £3.70 | |
| 2+ | | £14.75 | £0.00 | £11.05 | £3.70 | |

|  | **Aggregated costs** | | | | | | |
| --- | --- | --- | --- | --- | --- | --- | --- |
| Year | Graves Cost | Grave's proportion | Hashimoto's cost | Hashimoto's proportion | ‘Other’ ATE Cost | ‘Other' proportion | Aggregated Total |
| 1 | £1,275.73 | 0.63 | £345.01 | 0.15 | £330.26 | 0.22 | £928.12 |
| 2+ | £14.75 | 0.63 | £14.75 | 0.15 | 0 | 0.22 | £11.51 |

## 9. Risk of inhibitory anti-alemtuzumab antibodies

The risk of developing anti-alemtuzumab antibodies was sourced from a case series of 40 PwMS who were part of a longitudinal cohort in the UK (Wales) examining escalation vs early intensive disease-modifying therapy. This subset of 40 were those individuals in the longitudinal cohort who had been treated with alemtuzumab, and who had been administered at least 3 cycles. Of these 40 alemtuzumab-treated people, 32 had bioarchived serum samples which could be used to test for the presence of anti-drug antibodies [25].

Bioarchived serum sample data were available specifically from administration in cycle 3 for 31 of these. Of these, 4 failed to deplete lymphocytes by ≥ 35% following alemtuzumab administration in this cycle. Although not the primary outcome of this study, failure to deplete was associated with higher rates of disease activity in the 2 years following infusion than in those who demonstrated depletion [25].

This cohort were followed for all episodes of alemtuzumab administration (up to 6 cycles were received by some). In total 7 people saw limited depletion following alemtuzumab administration, and ADAs were present in 6 out of 7.

The mean proportion of individuals with neutralising antibodies was therefore estimated based on the proportion seeing limited depletion at cycle 3 combined with the proportion in which it was assumed ADAs were responsible for impaired alemtuzumab effect:

$$\bar{x}_{NAbs}=\frac{4}{31}\times\frac{6}{7}$$

$$\bar{x}_{NAbs}=0.1106$$

## 10. Parameters subject to probabilistic sensitivity analysis (PSA) with distributions used

| **Component** | **Distribution** |
| --- | --- |
| **EDSS progression in relapsing-remitting MS** | Dirichlet |
| **EDSS progression in secondary progressive MS** | Not varied in PSA |
| **Transition from relapsing-remitting MS to secondary progressive MS** | Not varied in PSA |
| **Mortality risk** | Background age and sex-specific mortality not varied  EDSS multipliers lognormal |
| **Annualised relapse rate in relapsing-remitting MS** | Logged coefficients drawn from normal distribution |
| **Disease-modifying therapy impact on annualised relapse rate** | Lognormal |
| **Disease-modifying therapy impact on EDSS progression** | Lognormal |
| **Health and Social Care costs** | EDSS-related costs: Gamma  Relapse-related costs: Gamma  DMT costs: scenario analysis undertaken varying price discount from 0-100% |
| **MS-related Quality of Life utility values** | EDSS-related QoL: Gamma  *Dis*utility of relapse: Gamma |
| **Disease modifying therapy intolerance** | Beta |
| **Progressive Multifocal Leukoencephalopathy risk** | Not varied in PSA |
| **Autoimmune thyroid disease risk** | Not varied in PSA |
| **Autoimmune thyroid disease cost** | Not varied in PSA |

## 11. Characteristics of Study Population

21 unique individual characteristic sets from a real-world population of individuals initiating alemtuzumab were included in the microsimulation model.

This data was sourced from Bart’s Health NHS Trust, which had a catchment population of over 2.5 million people, and provided tertiary care to people with MS in the United Kingdom. The population of people with MS under the care of Barts Health was approximately 3000, with approximately 1000 actively on DMT treatment pathways.

The characteristics of the individuals included in microsimulation were taken from records of people initiating alemtuzumab in the period 1^st^ January 2021 to 31^st^ March 2023 who had complete demographic and disease characteristics required in the model. This period was chosen to reflect current prescribing practice following a 2020 UK Medicines and Healthcare products Regulatory Agency safety update which was expected to have led to a more restrictive approach to prescribing [26].

In this period 40 people initiated alemtuzumab, 5.9% of DMT initiations, of whom 21 had sufficient information to provide individual characteristic sets for microsimulation modelling. Baseline characteristics in the simulated population were similar to the overall population including those with some missing information in relevant characteristics:

|  | **Microsimulation population**  **N=21** | **Total population**  **N=40** |
| --- | --- | --- |
|  | **Mean (Range) or N (%)** | **Mean (Range) or N (%)** |
| Female Sex | 13 (62%) | 32 (63%) |
| Age at MS onset | 30.7 (13 - 49) | 31.2 (13 - 49) |
| Age at alemtuzumab initiation | 36.9 (20-54) | 37.3 (20-67) |
| EDSS at alemtuzumab initiation | 3.48 (1-6) | 3.51 (1-6.5) |

# Supplemental Table 1. Model-projected incremental outcomes for individual study participants with alemtuzumab ADA testing strategy

|  | **Female  EDSS: 1 Age MS onset: 24 Age: 24** | **Male  EDSS: 1 Age MS onset: 35 Age: 41** | **Male  EDSS: 1 Age MS onset: 41 Age: 41** | **Female  EDSS: 2 Age MS onset: 20 Age: 20** | **Male  EDSS: 2 Age MS onset: 22 Age: 22** | **Male  EDSS: 2 Age MS onset: 30 Age: 30** |
| --- | --- | --- | --- | --- | --- | --- |
| Difference in total number of relapses | -0.0372 (0.0041) | -0.0288 (0.0034) | -0.0329 (0.0036) | -0.0326 (0.0038) | -0.0298 (0.0032) | -0.031 (0.0034) |
| Difference in time to SPMS | 0.1234 (0.0208) | 0.1203 (0.0167) | 0.1189 (0.0172) | 0.0972 (0.0169) | 0.0998 (0.0166) | 0.0992 (0.0153) |
| Difference in life-years | 0.0282 (0.0069) | 0.0485 (0.0079) | 0.048 (0.0082) | 0.0209 (0.0056) | 0.0279 (0.0064) | 0.0333 (0.0063) |
| Testing Costs | £23.14 (£0.03) | £23.14 (£0.03) | £23.15 (£0.03) | £22.47 (£0.03) | £22.47 (£0.03) | £22.47 (£0.04) |
| Difference in Total Costs | £3,734 (£526) | £4,841 (£374) | £4,702 (£422) | £2,991 (£417) | £3,283 (£355) | £3,452 (£345) |
| Difference in Total Costs (discounted) | £2,463 (£290) | £3,085 (£192) | £3,022 (£225) | £1,997 (£229) | £2,166 (£187) | £2,250 (£185) |
| Difference in Total QALYs | 0.116 (0.0192) | 0.1184 (0.016) | 0.1172 (0.0165) | 0.0953 (0.0159) | 0.0984 (0.0157) | 0.0989 (0.0147) |
| Difference in Total QALYs (discounted) | 0.0574 (0.0081) | 0.059 (0.0073) | 0.0593 (0.0075) | 0.048 (0.0067) | 0.0492 (0.0067) | 0.0497 (0.0065) |
| Incremental Cost per QALY gained  (with costs and QALYs discounted) | £45,508 (£5,281) | £54,622 (£5,495) | £53,177 (£5,513) | £44,493 (£5,197) | £47,063 (£5,049) | £45,675 (£5,084) |
| Difference in DMT Costs | £4,026 (£533) | £5,003 (£375) | £4,864 (£421) | £3,259 (£419) | £3,523 (£355) | £3,657 (£349) |
| Difference in EDSS-related Costs | £-306.96 (£107) | £-177.64 (£ 68.10) | £-177.29 (£ 67.66) | £-282.65 (£ 98.07) | £-255.27 (£ 88.60) | £-219.19 (£ 73.24) |
| Difference in Relapse-related Costs | £-9.10 (£1.04) | £-7.84 (£1.04) | £-8.75 (£1.05) | £-7.98 (£0.94) | £-7.56 (£0.89) | £-7.98 (£0.96) |
| Difference in Thyroid Adverse Event Costs | £0.12 (£0.03) | £0.20 (£0.03) | £0.20 (£0.03) | £0.09 (£0.02) | £0.11 (£0.03) | £0.14 (£0.03) |
| Difference in DMT Costs (discounted) | £2,602 (£293) | £3,170 (£190) | £3,109 (£224) | £2,125 (£230) | £2,285 (£187) | £2,357 (£185) |
| Difference in EDSS-related Costs (discounted) | £-153.59 (£46.86) | £-102.00 (£35.70) | £-102.95 (£35.99) | £-143.32 (£41.64) | £-135.12 (£39.72) | £-121.85 (£35.34) |
| Difference in Relapse-related Costs (discounted) | £-8.37 (£0.97) | £-6.33 (£0.89) | £-7.21 (£0.94) | £-7.27 (£0.89) | £-6.72 (£0.80) | £-6.86 (£0.87) |
| Difference in Thyroid Adverse Event Costs (discounted) | £0.03 (£0.01) | £0.07 (£0.01) | £0.07 (£0.01) | £0.02 (£0.01) | £0.03 (£0.01) | £0.04 (£0.01) |
| Difference in time on any DMT | 0.0825 (0.0206) | 0.1096 (0.0175) | 0.1069 (0.0184) | 0.0686 (0.0164) | 0.076 (0.0158) | 0.0813 (0.0154) |
| Difference in time on alemtuzumab | -0.2614 (0.0205) | -0.3203 (0.0163) | -0.3161 (0.0195) | -0.2181 (0.0154) | -0.2308 (0.0128) | -0.2385 (0.0135) |
| Difference in alemtuzumab cycles given | -0.0855 (0.0026) | -0.0757 (0.0032) | -0.0793 (0.003) | -0.0754 (0.0029) | -0.073 (0.0026) | -0.0728 (0.0026) |

**Supplemental Table 1 (cont).**  **Model-projected incremental outcomes for individual study participants with alemtuzumab ADA testing strategy**

|  | **Female  EDSS: 2 Age MS onset: 39 Age: 39** | **Male  EDSS: 2 Age MS onset: 39 Age: 41** | **Female  EDSS: 3 Age MS onset: 28 Age: 28** | **Female  EDSS: 3 Age MS onset: 32 Age: 33** | **Female  EDSS: 4 Age MS onset: 29 Age: 29** | **Female  EDSS: 4 Age MS onset: 32 Age: 33** |
| --- | --- | --- | --- | --- | --- | --- |
| Difference in total number of relapses | -0.0345 (0.0043) | -0.0285 (0.0031) | -0.0243 (0.0032) | -0.0245 (0.0035) | -0.0215 (0.0032) | -0.0218 (0.0034) |
| Difference in time to SPMS | 0.088 (0.0138) | 0.0893 (0.013) | 0.0555 (0.0094) | 0.0573 (0.0096) | 0.0411 (0.0073) | 0.0414 (0.0075) |
| Difference in life-years | 0.0302 (0.0057) | 0.0367 (0.0061) | 0.0162 (0.0037) | 0.0187 (0.0039) | 0.0128 (0.003) | 0.0144 (0.0031) |
| Testing Costs | £22.47 (£0.04) | £22.47 (£0.04) | £20.39 (£0.07) | £20.40 (£0.07) | £20.13 (£0.14) | £20.15 (£0.14) |
| Difference in Total Costs | £3,071 (£389) | £3,524 (£306) | £1,921 (£252) | £2,043 (£245) | £1,511 (£193) | £1,634 (£202) |
| Difference in Total Costs (discounted) | £2,041 (£225) | £2,337 (£169) | £1,296 (£143) | £1,370 (£141) | £1,038 (£112) | £1,113 (£119) |
| Difference in Total QALYs | 0.089 (0.0133) | 0.0912 (0.0126) | 0.0589 (0.0093) | 0.0612 (0.0094) | 0.0457 (0.0073) | 0.0466 (0.0075) |
| Difference in Total QALYs (discounted) | 0.0466 (0.0062) | 0.0479 (0.006) | 0.0308 (0.0041) | 0.0319 (0.0043) | 0.0243 (0.0034) | 0.0249 (0.0035) |
| Incremental Cost per QALY gained  (with costs and QALYs discounted) | £43,818 (£5,429) | £50,118 (£5,196) | £46,407 (£5,267) | £44,223 (£5,336) | £47,591 (£5,457) | £46,594 (£5,565) |
| Difference in DMT Costs | £3,244 (£393) | £3,665 (£310) | £2,071 (£251) | £2,185 (£248) | £1,629 (£194) | £1,745 (£204) |
| Difference in EDSS-related Costs | £-187.35 (£ 61.81) | £-156.11 (£ 54.83) | £-164.39 (£ 56.38) | £-156.06 (£ 52.54) | £-132.71 (£ 46.65) | £-125.26 (£ 43.11) |
| Difference in Relapse-related Costs | £-8.60 (£1.04) | £-7.52 (£0.93) | £-6.02 (£0.76) | £-6.17 (£0.84) | £-5.35 (£0.74) | £-5.51 (£0.82) |
| Difference in Thyroid Adverse Event Costs | £0.12 (£0.02) | £0.15 (£0.03) | £0.07 (£0.02) | £0.08 (£0.02) | £0.05 (£0.01) | £0.06 (£0.01) |
| Difference in DMT Costs (discounted) | £2,134 (£226) | £2,416 (£171) | £1,373 (£142) | £1,443 (£142) | £1,099 (£112) | £1,171 (£120) |
| Difference in EDSS-related Costs (discounted) | £-107.91 (£31.48) | £ -94.67 (£29.52) | £ -91.69 (£26.37) | £ -88.72 (£25.42) | £ -76.29 (£22.19) | £ -73.78 (£21.44) |
| Difference in Relapse-related Costs (discounted) | £-7.48 (£0.98) | £-6.32 (£0.82) | £-5.37 (£0.72) | £-5.35 (£0.78) | £-4.72 (£0.69) | £-4.74 (£0.75) |
| Difference in Thyroid Adverse Event Costs (discounted) | £0.04 (£0.01) | £0.05 (£0.01) | £0.02 (£0.00) | £0.02 (£0.00) | £0.02 (£0.00) | £0.02 (£0.00) |
| Difference in time on any DMT | 0.0709 (0.0147) | 0.0816 (0.0136) | 0.0452 (0.0094) | 0.0496 (0.0098) | 0.0365 (0.0072) | 0.0392 (0.0075) |
| Difference in time on alemtuzumab | -0.2234 (0.0168) | -0.2484 (0.0129) | -0.1497 (0.0093) | -0.1574 (0.0097) | -0.1257 (0.007) | -0.1324 (0.0081) |
| Difference in alemtuzumab cycles given | -0.0753 (0.0029) | -0.0707 (0.0027) | -0.0562 (0.0027) | -0.0561 (0.0029) | -0.05 (0.0031) | -0.0503 (0.0031) |

**Supplemental Table 1 (cont).**  **Model-projected incremental outcomes for individual study participants with alemtuzumab ADA testing strategy**

|  | **Female  EDSS: 4 Age MS onset: 29 Age: 38** | **Male  EDSS: 4 Age MS onset: 13 Age: 41** | **Female  EDSS: 4 Age MS onset: 33 Age: 46** | **Male  EDSS: 4 Age MS onset: 40 Age: 52** | **Male  EDSS: 6 Age MS onset: 29 Age: 35** |
| --- | --- | --- | --- | --- | --- |
| Difference in total number of relapses | -0.0164 (0.0028) | -0.0077 (0.0015) | -0.0125 (0.0025) | -0.0061 (0.0013) | -0.0082 (0.0014) |
| Difference in time to SPMS | 0.0422 (0.0073) | 0.0381 (0.0063) | 0.0404 (0.0066) | 0.0285 (0.0048) | 0.0093 (0.002) |
| Difference in life-years | 0.0162 (0.0032) | 0.0182 (0.0034) | 0.0185 (0.0032) | 0.0165 (0.0029) | 0.0052 (0.0012) |
| Testing Costs | £20.13 (£0.14) | £20.15 (£0.13) | £20.15 (£0.14) | £20.13 (£0.14) | £16.56 (£0.35) |
| Difference in Total Costs | £1,767 (£193) | £1,827 (£174) | £1,940 (£187) | £1,718 (£172) | £ 621 (£ 57.49) |
| Difference in Total Costs (discounted) | £1,195 (£111) | £1,196 (£105) | £1,285 (£108) | £1,139 (£108) | £ 441 (£ 35.14) |
| Difference in Total QALYs | 0.047 (0.0075) | 0.0424 (0.0066) | 0.0454 (0.0068) | 0.0331 (0.0052) | 0.0129 (0.0023) |
| Difference in Total QALYs (discounted) | 0.0248 (0.0035) | 0.0217 (0.0032) | 0.0239 (0.0033) | 0.0181 (0.0027) | 0.0072 (0.0012) |
| Incremental Cost per QALY gained  (with costs and QALYs discounted) | £53,066 (£5,500) | £59,398 (£5,831) | £57,525 (£5,712) | £67,102 (£6,397) | £79,920 (£7,692) |
| Difference in DMT Costs | £1,865 (£196) | £1,886 (£180) | £2,009 (£190) | £1,741 (£175) | £ 642 (£ 57.89) |
| Difference in EDSS-related Costs | £-113.62 (£ 38.60) | £ -76.36 (£ 28.79) | £ -85.39 (£ 29.43) | £ -41.85 (£ 17.36) | £ -35.28 (£ 14.17) |
| Difference in Relapse-related Costs | £-4.33 (£0.71) | £-2.16 (£0.43) | £-3.45 (£0.69) | £-1.75 (£0.38) | £-2.18 (£0.38) |
| Difference in Thyroid Adverse Event Costs | £0.07 (£0.01) | £0.07 (£0.01) | £0.07 (£0.01) | £0.07 (£0.01) | £0.02 (£0.01) |
| Difference in DMT Costs (discounted) | £1,246 (£112) | £1,225 (£109) | £1,321 (£110) | £1,149 (£111) | £ 450 (£ 35.52) |
| Difference in EDSS-related Costs (discounted) | £ -67.75 (£19.90) | £ -47.35 (£15.64) | £ -53.38 (£16.24) | £ -28.91 (£10.39) | £ -23.49 (£ 7.84) |
| Difference in Relapse-related Costs (discounted) | £-3.68 (£0.64) | £-1.78 (£0.38) | £-2.83 (£0.59) | £-1.40 (£0.32) | £-1.83 (£0.33) |
| Difference in Thyroid Adverse Event Costs (discounted) | £0.02 (£0.00) | £0.03 (£0.01) | £0.03 (£0.00) | £0.03 (£0.01) | £0.01 (£0.00) |
| Difference in time on any DMT | 0.0418 (0.0075) | 0.0417 (0.0065) | 0.0452 (0.007) | 0.0354 (0.0054) | 0.0139 (0.0022) |
| Difference in time on alemtuzumab | -0.1359 (0.0085) | -0.1303 (0.0102) | -0.1432 (0.0099) | -0.1275 (0.0115) | -0.0578 (0.0037) |
| Difference in alemtuzumab cycles given | -0.0451 (0.0033) | -0.0327 (0.0032) | -0.0398 (0.0034) | -0.0292 (0.0031) | -0.0235 (0.002) |

**Supplemental Table 1 (cont).**  **Model-projected incremental outcomes for individual study participants with alemtuzumab ADA testing strategy**

|  | **Female  EDSS: 6 Age MS onset: 21 Age: 41** | **Female  EDSS: 6 Age MS onset: 39 Age: 41** | **Female  EDSS: 6 Age MS onset: 21 Age: 46** | **Female  EDSS: 6 Age MS onset: 49 Age: 54** |
| --- | --- | --- | --- | --- |
| Difference in total number of relapses | -0.0043 (0.0011) | -0.0112 (0.0021) | -0.0031 (8e-04) | -0.0062 (0.0015) |
| Difference in time to SPMS | 0.0097 (0.0022) | 0.0083 (0.0019) | 0.0089 (0.0019) | 0.0072 (0.0015) |
| Difference in life-years | 0.0051 (0.0012) | 0.0048 (0.0012) | 0.005 (0.0011) | 0.0049 (0.001) |
| Testing Costs | £16.45 (£0.38) | £16.55 (£0.35) | £16.45 (£0.38) | £16.56 (£0.35) |
| Difference in Total Costs | £ 627 (£ 72.03) | £ 607 (£ 64.79) | £ 608 (£ 73.25) | £ 604 (£ 53.39) |
| Difference in Total Costs (discounted) | £ 430 (£ 44.74) | £ 438 (£ 40.59) | £ 414 (£ 46.47) | £ 430 (£ 34.16) |
| Difference in Total QALYs | 0.0126 (0.0025) | 0.0125 (0.0022) | 0.0115 (0.0022) | 0.0104 (0.0017) |
| Difference in Total QALYs (discounted) | 0.0066 (0.0012) | 0.0072 (0.0011) | 0.0061 (0.0011) | 0.0061 (0.001) |
| Incremental Cost per QALY gained  (with costs and QALYs discounted) | £66,596 (£7,879) | £75,655 (£8,162) | £72,549 (£8,050) | £92,818 (£8,596) |
| Difference in DMT Costs | £ 644 (£ 74.45) | £ 629 (£ 64.27) | £ 617 (£ 75.91) | £ 610 (£ 53.83) |
| Difference in EDSS-related Costs | £ -31.93 (£ 12.93) | £ -35.46 (£ 14.01) | £ -25.13 (£ 10.24) | £ -20.90 (£ 7.82) |
| Difference in Relapse-related Costs | £-1.20 (£0.30) | £-2.89 (£0.52) | £-0.88 (£0.23) | £-1.71 (£0.40) |
| Difference in Thyroid Adverse Event Costs | £0.02 (£0.01) | £0.02 (£0.01) | £0.02 (£0.00) | £0.02 (£0.00) |
| Difference in DMT Costs (discounted) | £ 435 (£ 46.53) | £ 447 (£ 40.23) | £ 415 (£ 48.34) | £ 430 (£ 34.60) |
| Difference in EDSS-related Costs (discounted) | £ -20.36 (£ 7.18) | £ -24.03 (£ 8.00) | £ -16.43 (£ 5.90) | £ -15.08 (£ 5.03) |
| Difference in Relapse-related Costs (discounted) | £-0.99 (£0.26) | £-2.45 (£0.46) | £-0.72 (£0.20) | £-1.41 (£0.35) |
| Difference in Thyroid Adverse Event Costs (discounted) | £0.01 (£0.00) | £0.01 (£0.00) | £0.01 (£0.00) | £0.01 (£0.00) |
| Difference in time on any DMT | 0.0141 (0.0024) | 0.014 (0.0023) | 0.0131 (0.0023) | 0.0124 (0.0018) |
| Difference in time on alemtuzumab | -0.0519 (0.0051) | -0.0606 (0.0035) | -0.0487 (0.0055) | -0.0552 (0.0043) |
| Difference in alemtuzumab cycles given | -0.0171 (0.0022) | -0.0271 (0.0023) | -0.0149 (0.002) | -0.0204 (0.0024) |

**REFERENCES**

1. Palace, J., Bregenzer, T., Tremlett, H., Oger, J., Zhu, F., Boggild, M., Duddy, M., Dobson, C.: UK multiple sclerosis risk-sharing scheme: a new natural history dataset and an improved Markov model. BMJ Open. 4, e004073 (2014). https://doi.org/10.1136/bmjopen-2013-004073

2. Mauskopf, J., Fay, M., Iyer, R., Sarda, S., Livingston, T.: Cost-effectiveness of delayed-release dimethyl fumarate for the treatment of relapsing forms of multiple sclerosis in the United States. J. Med. Econ. 19, 432–442 (2016). https://doi.org/10.3111/13696998.2015.1135805

3. Scalfari, A., Neuhaus, A., Degenhardt, A., Rice, G.P., Muraro, P.A., Daumer, M., Ebers, G.C.: The natural history of multiple sclerosis: a geographically based study 10: relapses and long-term disability. Brain. 133, 1914–1929 (2010). https://doi.org/10.1093/brain/awq118

4. United Kingdom Office For National Statistics,: National Life Tables: UK, https://www.ons.gov.uk/peoplepopulationandcommunity/birthsdeathsandmarriages/lifeexpectancies/datasets/nationallifetablesunitedkingdomreferencetables, (2021)

5. Zimmermann, M., Brouwer, E., Tice, J.A., Seidner, M., Loos, A.M., Liu, S., Chapman, R.H., Kumar, V., Carlson, J.J.: Disease-Modifying Therapies for Relapsing–Remitting and Primary Progressive Multiple Sclerosis: A Cost-Utility Analysis. CNS Drugs. 32, 1145–1157 (2018). https://doi.org/10.1007/s40263-018-0566-9

6. Tremlett, H., Zhao, Y., Joseph, J., Devonshire, V., UBCMS Clinic Neurologists: Relapses in multiple sclerosis are age- and time-dependent. J. Neurol. Neurosurg. Psychiatry. 79, 1368–1374 (2008). https://doi.org/10.1136/jnnp.2008.145805

7. Scappaticcio, L., Castellana, M., Virili, C., Bellastella, G., Centanni, M., Cannavò, S., Campennì, A., Ruggeri, R.M., Giovanella, L., Trimboli, P.: Alemtuzumab-induced thyroid events in multiple sclerosis: a systematic review and meta-analysis. J. Endocrinol. Invest. 43, 219–229 (2020). https://doi.org/10.1007/s40618-019-01105-7

8. Medicines and Healthcare products Regulatory Agency (MHRA): Natalizumab (Tysabri▼): progressive multifocal leukoencephalopathy—updated advice to support early detection. (2016)

9. Bozic, C., Subramanyam, M., Richman, S., Plavina, T., Zhang, A., Ticho, B.: Anti-JC virus (JCV) antibody prevalence in the JCV Epidemiology in MS (JEMS) trial. Eur. J. Neurol. 21, 299–304 (2014). https://doi.org/10.1111/ene.12304

10. Dong-Si, T., Richman, S., Wattjes, M.P., Wenten, M., Gheuens, S., Philip, J., Datta, S., McIninch, J., Bozic, C., Bloomgren, G., Richert, N.: Outcome and survival of asymptomatic PML in natalizumab-treated MS patients. Ann. Clin. Transl. Neurol. 1, 755–764 (2014). https://doi.org/10.1002/acn3.114

11. Hennessy, B., Zierhut, M.L., Kracker, H., Keenan, A., Sidorenko, T.: Comparative efficacy of relapsing multiple sclerosis therapies: Model-based meta-analyses for confirmed disability accumulation and annualized relapse rate. Mult. Scler. Relat. Disord. 64, 103908 (2022). https://doi.org/10.1016/j.msard.2022.103908

12. Hillert, J., Magyari, M., Soelberg Sørensen, P., Butzkueven, H., Van Der Welt, A., Vukusic, S., Trojano, M., Iaffaldano, P., Pellegrini, F., Hyde, R., Stawiarz, L., Manouchehrinia, A., Spelman, T.: Treatment switching and discontinuation over 20 years in the Big multiple sclerosis data network. Front. Neurol. 12, 647811 (2021). https://doi.org/10.3389/fneur.2021.647811

13. Joint Formulary Committee: BNF 84 (British National Formulary) September 2022: 84: September 2022 - March 2023. Pharmaceutical Press (2022)

14. electronic medicines compendium (emc), https://www.medicines.org.uk/emc/

15. Hawton, A.J., Green, C.: Multiple sclerosis: relapses, resource use, and costs. Eur. J. Health Econ. 17, 875–884 (2016). https://doi.org/10.1007/s10198-015-0728-3

16. Thompson, A., Kobelt, G., Berg, J., Capsa, D., Eriksson, J., Miller, D., European Multiple Sclerosis Platform: New insights into the burden and costs of multiple sclerosis in Europe: Results for the United Kingdom. Mult. Scler. 23, 204–216 (2017). https://doi.org/10.1177/1352458517708687

17. Heather, A., Goodwin, E., Green, C., Morrish, N., Ukoumunne, O.C., Middleton, R.M., Hawton, A.: Multiple sclerosis health-related quality of life utility values from the UK MS register. Mult. Scler. J. Exp. Transl. Clin. (2023)

18. Hawton, A., Green, C.: Health utilities for multiple sclerosis. Value Health. 19, 460–468 (2016). https://doi.org/10.1016/j.jval.2016.01.002

19. Sadovnick, A.D., Ebers, G.C., Wilson, R.W., Paty, D.W.: Life expectancy in patients attending multiple sclerosis clinics. Neurology. 42, 991–994 (1992). https://doi.org/10.1212/wnl.42.5.991

20. Pokorski, R.J.: Long-term survival experience of patients with multiple sclerosis. J. Insur. Med. 29, 101–106 (1997)

21. NHS England: National Schedule of NHS Costs, https://www.england.nhs.uk/costing-in-the-nhs/national-cost-collection/#ncc1819, (2021)

22. NHS Business Services Authority: Drug Tariff, (2022)

23. Ragavan, S., Elhelw, O., Majeed, W., Kyriacou, A., Syed, A.: Alemtuzumab-Induced Autoimmune Thyroid Dysfunction. Cureus. 14, e22751 (2022). https://doi.org/10.7759/cureus.22751

24. Burch, H.B., Cooper, D.S.: Management of Graves Disease: A Review. JAMA. 314, 2544–2554 (2015). https://doi.org/10.1001/jama.2015.16535

25. Saxena, G., Moore, J.M., Jones, M., Pryce, G., Ali, L., Leisegang, G.R., Vijay, V., Loveless, S., Robertson, N.P., Schmierer, K., Giovannoni, G., Gnananpavan, S., Baker, D., Tallantyre, E.C., Kang, A.S.: Detecting and predicting neutralization of alemtuzumab responses in MS. Neurol. Neuroimmunol. Neuroinflamm. 7, e767 (2020). https://doi.org/10.1212/NXI.0000000000000767

26. Medicines and Healthcare products Regulatory Agency: Lemtrada (alemtuzumab): updated restrictions and strengthened monitoring requirements following review of serious cardiovascular and immune-mediated reactions. (2020)
